# Brainstem circuit for sickness-induced sleep

**DOI:** 10.1126/sciadv.ady0245

**Published:** 2025-12-10

**Authors:** Dana Darmohray, Jiao Sima, Chien-Hao Chen, Daniel Silverman, Changwan Chen, Ashley Xu, Yuanyuan Yao, Yang Dan

**Affiliations:** ^1^ Department of Neuroscience, Helen Wills Neuroscience Institute, Howard Hughes Medical Institute, University of California, Berkeley, Berkeley, CA 94720, USA.; ^2^ Institute of Neuromodulation and Cognition (INC), Shenzhen Medical Academy of Research and Translation (SMART), Shenzhen 518107, Guangdong, China.

## Abstract

Increased sleep induced by immune activation plays a crucial role in facilitating recovery from illness. However, the neural mechanisms underlying sickness-induced sleep remain poorly understood. Here, we identify a brainstem circuit originating in the nucleus of the solitary tract (NST) that mediates sickness-induced nonrapid eye movement (NREM) sleep. Using activity-dependent genetic labeling, we tagged NST neurons activated by lipopolysaccharide (LPS) injection and showed that their chemogenetic activation strongly promotes NREM sleep. These NST neurons project extensively to the parabrachial nucleus (PB), where LPS-activated neurons also promote NREM sleep. Fiber photometry recording of several wake-promoting neuromodulators using their biosensors showed that evoked norepinephrine release from locus coeruleus neurons is markedly reduced by either LPS injection or direct activation of NST or PB sickness neurons. These results suggest that sickness-induced NREM sleep is mediated in part by a brainstem circuit that regulates neuromodulator signaling.

## Teaser

Brainstem circuit mediates sickness-induced NREM sleep.

## INTRODUCTION

Sleep is a highly conserved innate behavior that is indispensable for health and survival. It supports various cognitive and physiological processes, including memory consolidation, emotional processing, waste clearance, and metabolic regulation (*1*–*6*). Sleep also interacts bidirectionally with the immune system (*7*–*11*). Sleep loss leads to immune system dysregulation and ultimately death (*12*–*14*). Conversely, following an immune challenge, the amount of time the animal spends in sleep increases substantially (*15*, *16*). Such an increase in sleep promotes functional recovery and survival during sickness or injury (*7*, *17*, *18*). However, the mechanisms by which the immune system regulates sleep are not well understood.

A range of sickness-induced behavioral changes, collectively known as sickness behavior, can be triggered by cytokines—signaling molecules that play crucial roles in regulating immune responses (*19*–*22*). Cytokine signals in the periphery can reach the brain through neural and humoral routes. The neural pathway begins with the sensory division of the vagus nerve, which projects to the nucleus of the solitary tract (NST) in the dorsal medulla. Vagotomy or reversible inactivation of the NST strongly abrogates sickness behavior, indicating a crucial role of this pathway in mediating the immune-brain communication (*23*–*27*). Recent studies have yielded important insights into how this pathway controls sickness behavior by identifying the subsets of vagal sensory neurons that sense peripheral inflammation and the NST neurons that drive sickness behavior (*24*, *28*).

While these studies elucidate the neural entry point for sickness behavior in general, the downstream pathways mediating specific symptoms such as increased sleep remain unclear. The NST is widely interconnected with brain areas that regulate functions spanning autonomic outflow to motivated behavior (*29*–*35*), allowing it to orchestrate multiple physiological and behavioral responses to sickness. Here, we explore the neural pathways from the NST that drive sleep during peripheral immune activation. Using activity-dependent genetic labeling and chemogenetic manipulation, we show that sickness-activated NST neurons and their projection target—the parabrachial nucleus (PB)—can promote nonrapid eye movement (NREM) sleep in the absence of inflammation. Using genetically encoded fluorescence-based GPCR activation-based (GRAB) sensors for several wake-promoting neuromodulators, we show that evoked norepinephrine (NE) release from the locus coeruleus (LC) is markedly reduced by peripheral inflammation or direct activation of NST or PB sickness neurons. These results suggest that sickness-induced NREM sleep could be mediated in part by a brainstem circuit that regulates neuromodulator signaling.

## RESULTS

### NST sickness-activated neurons promote NREM sleep

**Fig. 1.**
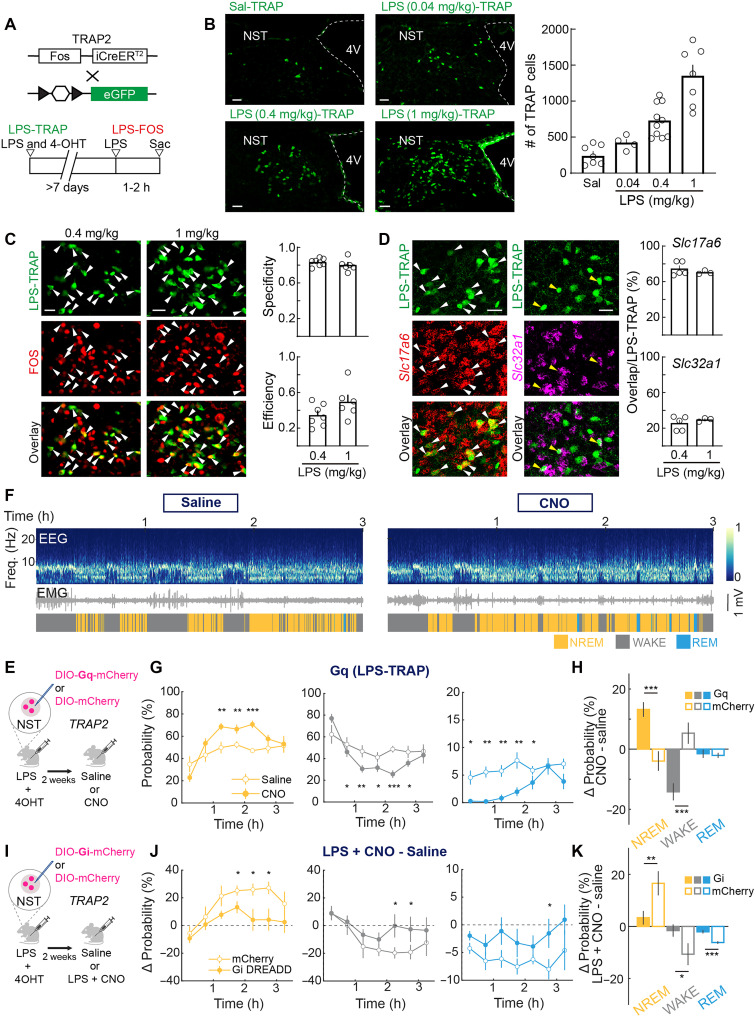
NST sickness-activated neurons promote NREM sleep. (**A**) TRAP2 strategy for labeling sickness-activated neurons. TRAP2 (Fos-2A-iCreER^T2^) crossing with Cre-inducible eGFP or tdTomato reporter mice received tamoxifen (4-OHT, 20 mg/kg) with LPS to tag sickness-responsive NST neurons (LPS-TRAP). After >7 days, a second LPS injection was used for Fos labeling (LPS-FOS). (**B**) Left: Examples of eGFP/tdTomato-labeled NST neurons in Sal-TRAP and LPS-TRAP conditions. Right: Quantification of TRAP cells across groups. Scale bar, 50 μm. (**C**) Overlap of LPS-TRAP (green) and LPS-FOS (red) neurons at 0.4 and 1 mg/kg. Colabeled cells indicated by arrows. Right: Quantification of specificity (overlap/LPS-TRAP) and efficiency (overlap/LPS-FOS). Scale bar, 25 μm. (**D**) Overlap between LPS-TRAP neurons and cell type markers *Slc17a6* and *Slc32a1*. Right: Quantification (means ± SEM). Open circles, individual samples. Scale bar, 25 μm. (**E**) Chemogenetic activation design. LPS and 4-OHT were injected into TRAP2 mice to induce the expression of either hM3D(G_q_)-mCherry or mCherry in NST^LPS-TRAP^ neurons. Two weeks later, the effect of saline or CNO on sleep was measured. Created in BioRender. Darmohray, D. (2025) https://BioRender.com/wd76zgb. (**F**) Example EEG/EMG traces and state scoring following saline (left) or CNO (right) injection. h, hours. (**G**) Average changes in NREM, wake, and REM following saline (open circles) or CNO injection (solid circles, *n* = 10) in TRAP2 mice expressing hM3D(G_q_). Tukey’s post hoc, *P < 0.05, **P < 0.01, ***P < 0.001. (**H**) State differences between saline and CNO conditions in hM3D(G_q_) (*n* = 10) and mCherry controls (*n* = 8). Independent samples *t* tests, *P < 0.05, **P < 0.01, ***P < 0.001. (**I**) Chemogenetic inhibition design. hM4D(G_i_)-mCherry or mCherry was expressed in NST^LPS-TRAP^ neurons. (**J**) Brain state changes between LPS + CNO and saline in hM4D(G_i_) (solid, *n* = 7) and mCherry (open, *n* = 7) groups. Statistics as in (G). (**K**) Summary of saline versus LPS + CNO effects in hM4D(G_i_) and mCherry groups. Statistics as in (H).

To elucidate the neural circuitry underlying sickness-induced sleep, we first tested whether stimulating sickness-activated NST neurons is sufficient to increase sleep in the absence of peripheral immune activation. We used activity-dependent genetic labeling (*36*) to tag sickness-activated neurons in the NST. TRAP2 mice expressing an inducible Cre (2A-iCreER^T2^) under the *Fos* promoter were crossed to a reporter line expressing enhanced green fluorescent protein (eGFP) or tdTomato (Fig. 1A). Lipopolysaccharide (LPS) was injected intraperitoneally (ip) to elicit sickness behavior together with tamoxifen [4-hydroxytamoxifen (4-OHT) 20 mg/kg]. After >7 days, we found more eGFP/tdTomato-labeled neurons in the NST (referred to as “NST^LPS-TRAP^” neurons) compared to saline-injected control mice, and the number of labeled neurons increased as the LPS dose was raised from 0.04 to 1 mg/kg (Fig. 1B). Immunohistochemical staining of FOS confirmed LPS-induced activation of NST neurons, and comparison of FOS and eGFP/tdTomato expression showed high specificity (0.4 mg/kg: 83.3% ± 1.7%;1 mg/kg: 79.6% ±2.8%, SEM) and moderate efficiency (0.4 mg/kg: 34.4% ± 4.7%; 1 mg/kg: 48.8% ± 7.6%, SEM) of LPS-TRAP labeling (Fig. 1C). Fluorescent in situ hybridization (FISH) showed that 74.5 ± 3.4% (means ± SEM) of NST^LPS-TRAP^ (0.4 mg/kg) neurons expressed the glutamatergic marker *Slc17a6* (encoding vesicular glutamate transporter 2), while 25.5 ± 3.5% expressed the GABAergic/glycinergic marker *Slc32a1* (encoding vesicular GABA transporter) (Fig. 1D; 1 mg/kg: 70.4 ± 1.2% glutamatergic, 29.6 ± 1.2% GABAergic/glycinergic).

We then tested the effect of chemogenetic activation of NST^LPS-TRAP^ neurons (Fig. 1E). Sleep-wake states were measured in freely moving mice in their home cage, based on electroencephalogram (EEG) and electromyogram (EMG) recordings (Fig. 1F). In mice expressing excitatory DREADD [hM3D(G_q_)-mCherry] in NST^LPS-TRAP^ neurons, clozapine-*N*-oxide (CNO, 0.3 mg/kg, ip) injection caused a strong increase in NREM sleep and reduction in wakefulness and REM sleep compared to saline control [Fig. 1, F and G; repeated measures analysis of variance (ANOVA); WAKE: *F*_(6,103)_ = 4.84, *P* = 0.0002; NREM: *F*_(6,107)_ = 4.59, *P* = 0.0003, REM: *F*_(6,95)_ = 2.98, *P* = 0.01]. In control mice expressing mCherry without hM3D(G_q_), CNO had no effect, and the changes in brain states induced by CNO were significantly different between hM3D(G_q_) and control mice [Fig. 1H; independent samples *t* test: WAKE: *t*_(16)_ = −3.39, *P* = 0.004; NREM: *t*_(16)_ = 4.21, *P* = 0.0006, REM: *t*_(16)_ = −1.13, *P* = 0.27]. Thus, activating NST^LPS-TRAP^ neurons is sufficient to promote NREM sleep, similar to the effects of LPS or Poly(I:C) injection [fig. S1, A to E; repeated measures ANOVA; 0.4 mg/kg LPS: WAKE: *F*_(1,104)_ = 7.50, *P* = 0.007; NREM: *F*_(1,104)_ = 27.45, *P* = 8.5 × 10^−07^; REM: *F*_(6,104)_ = 4.39, *P* = 0.0005; 20 mg/kg Poly(I:C): WAKE: *F*_(6,161)_ = 2.17, *P* = 0.05; NREM: *F*_(6,160)_ = 3.57, *P* = 0.002; REM: *F*_(1,158)_ = 78.03, *P* = 1.8 × 10^−15^].

We next tested whether the activity of NST^LPS-TRAP^ neurons is required for the LPS-induced increase in NREM sleep using chemogenetic inactivation. In mice expressing the inhibitory DREADD [hM4D(G_i_)-mCherry] in NST^LPS-TRAP^ neurons, but not in control mice expressing mCherry only, CNO (1 mg/kg, ip) injection significantly attenuated the LPS-induced increase in NREM sleep [Fig. 1, I and J; independent samples ANOVA; WAKE: *F*_(1,94)_ = 7.85, *P* = 0.006; NREM: *F*_(1,94)_ = 20.73, *P* = 1.5e-05; REM: *F*_(1,94)_ = 15.59, *P* = 0.0001], and the difference between hM4D(G_i_) and control mice was significant [Fig. 1K, independent samples *t* test; WAKE: *t*_(12)_ = 2.60, *P* = 0.02; NREM: *t*_(12)_ = −3.87, *P* = 0.002, REM: *t*_(12)_ = 2.15, *P* = 0.05].

**Fig. 2.**
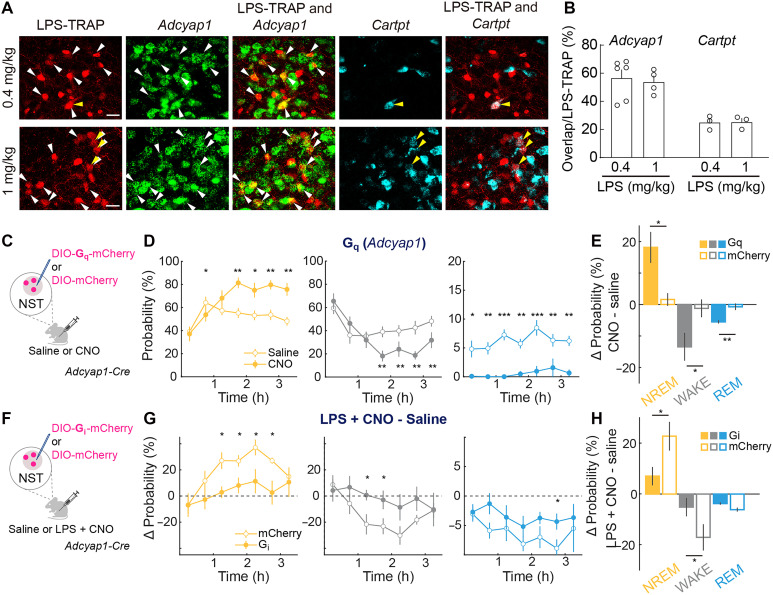
NST Adcyap1 neurons promote NREM sleep. (**A**) Example histological images showing overlap between LPS-TRAP (0.4 and 1 mg/kg) neurons and cell type markers, *Adcyap1* and *Cartpt* shown by double FISH. Arrows indicate colabeled neurons. Scale bar, 25 μm. (**B**) Quantification of the proportion of LPS-TRAP neurons coexpressing *Adcyap1* or *Cartpt* (means ± SEM). Open circles represent individual samples. (**C**) Experimental design for chemogenetic activation. Created in BioRender. Darmohray, D. (2025) https://BioRender.com/wd76zgb. (**D**) Average changes in NREM, wake, and REM following saline (open circles) or CNO injection (solid circles, *n* = 8) in *Adcyap1*-*Cre* mice expressing hM3D(G_q_). Horizontal axis represents time after injection. Asterisks indicate *P* values from Tukey’s post hoc test where *P < 0.05, **P < 0.01, ***P < 0.001. Error bars, ± SEM. (**E**) Differences in brain states between saline and CNO conditions in hM3D(G_q_) (*n* = 8) and mCherry control (*n* = 7) mice. Error bars, SEM. Asterisks indicate *P* values for independent samples *t* tests, where *P < 0.05, **P < 0.01, ***P < 0.001. (**F**) Experimental design for chemogenetic inhibition. Created in BioRender. Darmohray, D. (2025) https://BioRender.com/wd76zgb. (**G**) Differences in brain states between LPS + CNO and saline in hM4D(G_i_) (solid, *n* = 6) and mCherry (open, *n* = 6) groups. Horizontal axis represents time after injection. Statistics as in (D). (**H**) Difference in brain states between saline and LPS + CNO conditions for hM4D(G_i_) (*n* = 6) and mCherry (*n* = 6) groups. Error bars, SEM. Statistics as in (E).

A previous study showed that multiple aspects of LPS-induced sickness behavior were controlled by NST neurons expressing *Adcyap1* (encoding pituitary adenylate cyclase–activating polypeptide) (*24*), whereas another study showed that neurons expressing *Cartpt* (encoding cocaine- and amphetamine-regulated transcript) promote sleep in addition to cardiovascular regulation (*31*). We calculated the overlap of NST^LPS-TRAP^ neurons with *Adcyap1^+^* and *Cartpt*^+^ neurons (Fig. 2, A and B, and fig. S2A). The majority of NST^LPS-TRAP^ neurons (0.4 mg/kg: 56.7 ± 5.8%) expressed *Adcyap1*, and only a small fraction (24.6 ± 2.9%) expressed *Cartpt* (Fig. 2, A and B). Chemogenetic activation of *Adcyap1*-expressing NST neurons (NST*^Adcyap1^*) increased NREM sleep while reducing both wakefulness and REM sleep [Fig. 2, C and D, repeated measures ANOVA; WAKE: *F*_(6,78)_ = 3.42, *P* = 0.005; NREM: *F*_(6,78)_ = 6.05, *P* = 3.1 × 10^−05^; REM: *F*_(1,78)_ = 129.60, *P* = 2 × 10^−16^], an effect not observed in control mice expressing only mCherry in NST*^Adcyap1^* neurons [Fig. 2E; independent samples *t* test: WAKE: *t*_(13)_ = −2.79, *P* = 0.01; NREM: *t*_(13)_ = 3.26, *P* = 0.006, REM: *t*_(13)_ = −5.05, *P* = 2.2 × 10^−04^]. In mice expressing hM4D(G_i_)-mCherry in NST*^Adcyap1^* neurons, but not in control mice expressing mCherry only, CNO (1 mg/kg, ip) injection significantly attenuated the LPS-induced increase in NREM sleep [Fig. 2, F and G, independent samples ANOVA: WAKE: *F*_(1,77)_ = 10.77, *P* = 0.002; NREM: *F*_(1,77)_ = 19.92, *P* = 0.00003; REM: *F*_(1,77)_ = 6.10, *P* = 0.02], and the difference between hM4D(G_i_) and control mice was significant [Fig. 2H; WAKE: *t*_(10)_ = 3.45, *P* = 0.006; NREM: *t*_(10)_ = −4.32, *P* = 0.002; REM: *t*_(10)_ = 1.33, *P* = 0.2]. These results are consistent with chemogenetic activation and inactivation of NST^LPS-TRAP^ neurons (Fig. 1, E to K).

### NST-to-PB projection promotes NREM sleep

To identify the downstream pathways by which NST sickness-activated neurons promote sleep, we traced their axonal projections by injecting AAV8-pCAG-FLEX-eGFP into the NST of *Adcyap1*-*Cre* mice (fig. S3A). eGFP-labeled axons were observed in multiple brain areas, including the bed nucleus of the stria terminalis (BNST), several hypothalamic and thalamic regions, periaqueductal gray (PAG), amygdala (AMY), LC, PB, and several other brainstem areas (fig. S3B).

**Fig. 3.**
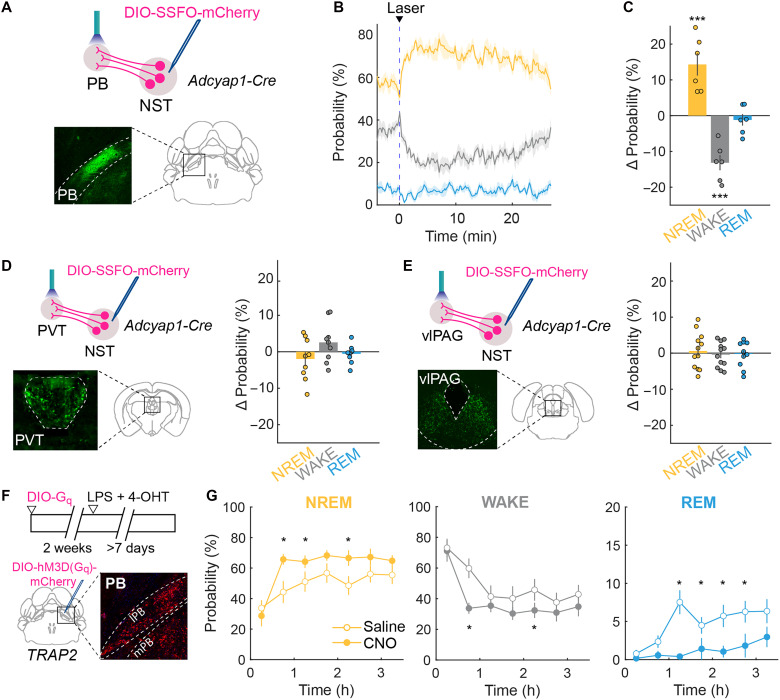
NST-to-PB projection promotes NREM sleep. (**A**) Experimental design for optogenetic activation of NST → PB terminals. Adcyap1-Cre mice were injected with AAV5-EF1a-DIO-SSFO-mCherry in the NST, and optic fibers were implanted above PB. During each 4-hour session, a 5-s laser pulse was delivered every 20 ± 5 min. Inset, axons from NST*^Adcyap1^* neurons terminating in PB. (**B**) Laser-evoked changes in brain states (NREM, wake, and REM) during NST ➔ PB terminal stimulation. Line represents average across individual mice (*n* = 6); shading represents ±SEM. Vertical blue line marks laser onset. (**C**) Quantification of laser-evoked brain-state changes in the 20 min following laser onset relative to 5 min baseline. Circles represent individual animals. Error bars, ±SEM. Asterisks indicate *P* values for paired samples *t* tests, where ***P < 0.001. (**D**) Left: Experimental design as in (A) but for NST➔PVT terminals. Inset, axon terminals from NST*^Adcyap1^* neurons terminating in PVT. Right: Laser-evoked changes in brain states (*n* = 9). (**E**) Left: Experimental design as in (A) but for NST➔vlPAG terminals. Inset, axon terminals from NST*^Adcyap1^* neurons terminating in vlPAG. Right: Laser-evoked changes in brain states (*n* = 12). (**F**) Top: Schematic of PB^LPS-TRAP^ experimental protocol. TRAP2 mice were injected with DIO-hM3D(G_q_) in PB. Two weeks later, LPS (0.4 mg/kg) and tamoxifen (4-OHT; 20 mg/kg) were coinjected to induce G_q_ expression in PB^LPS-TRAP^ neurons. Bottom: Coronal diagram showing PB injection site in TRAP2 mice. Inset, example fluorescence image of mCherry expression in PB. (**G**) Brain-state changes following saline (open circles) or CNO injection (solid circles, *n* = 9) in TRAP2 mice expressing hM3D(G_q_). Horizontal axis represents time after injection. Asterisks indicate *P* values from Tukey’s post hoc test where *P < 0.05. Error bars, ±SEM.

We then selected brain areas with strong NST*^Adcyap1^* projections and known roles in regulating brain states for functional investigation. We expressed a stabilized step function opsin (SSFO; AAV5-EF1a-DIO-SSFO-mCherry) (*37*) in NST*^Adcyap1^* neurons and optogenetically stimulated their axon terminals (6 mW, 5-s duration, applied every 20 ± 5 min) in the PB (Fig. 3A), paraventricular thalamus (PVT; Fig. 3D), and ventrolateral periaqueductal gray (vlPAG; Fig. 3E). Activation of the NST➔PB projection caused a significant increase in NREM sleep and reduction in wakefulness [Fig. 3, B and C; repeated measures ANOVA; *F*_(2,30)_ = 30.1, *P* = 0.00006; laser – baseline, wake: *t*_(5)_ = 6.12, *P* = 0.001; NREM: *t*_(5)_ = −4.09, *P* = 0.009; REM: *t*_(5)_ = 0.74, *P* = 0.5]. In contrast, stimulation of NST➔PVT and NST➔vlPAG projections caused no significant change in brain states [Fig. 3, D and E; repeated measures ANOVA; PVT, *F*_(2,24)_ = 0.9, *P* = 0.4; vlPAG, *F*_(2,30)_ = 0.6, *P* = 0.6].

To further examine the role of the PB in sickness-induced sleep, we tagged LPS-activated neurons in the PB using TRAP2 mice (Fig. 3F). Chemogenetic activation of PB^LPS-TRAP^ neurons markedly increased NREM sleep and reduced wakefulness and REM sleep [Fig. 3G; repeated measures ANOVA; WAKE: *F*_(1,91)_ = 12.88, *P* = 0.0005; NREM: *F*_(1,91)_ = 19.5, *P* = 2.8 × 10^−05^; REM: *F*_(6,91)_ = 2.4, *P* = 0.04], consistent with the effect of activating NST*^Adcyap1^* ➔ PB terminals.

### LPS-induced changes in neuromodulatory systems

**Fig. 4.**
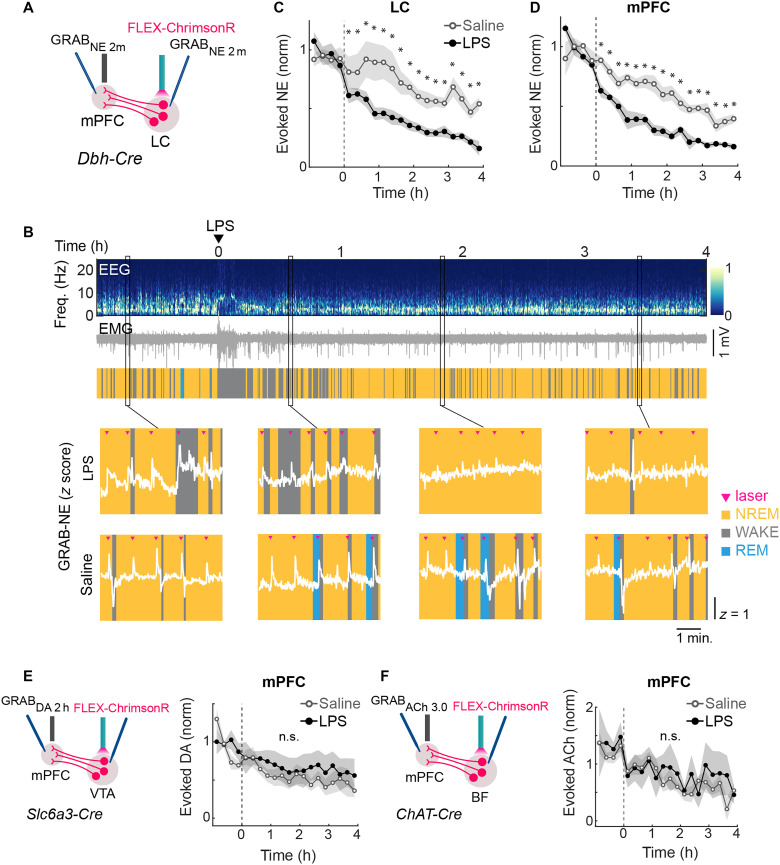
LPS-induced changes in neuromodulatory systems. (**A**) Schematic of protocol to measure laser-evoked neurotransmitter release in noradrenergic (NE) neurons. (**B**) Top: Example session showing EEG, EMG, and color-coded brain states during LPS administration. Bottom: LC GRAB_NE *z*-scored fluorescence traces overlaid on brain states before and after LPS or saline injection. Pink arrowheads indicate laser pulses used to evoke NE release. (**C**) Laser-evoked GRAB_NE_ response in LC, averaged in 15-min bins (*n* = 12). Horizontal axis indicates time from LPS (black) or saline (gray) injection. Lines show group average; shading represents ±SEM. Asterisks indicate *P* values from Tukey’s post hoc test (*P < 0.05). (**D**) The same as (C) but for mPFC (*n* = 7). (**E**) Left: Schematic for optogenetic measurement of laser-evoked dopaminergic (DA) release. Right: GRAB_DA response in mPFC, averaged in 15-min bins (*n* = 6). Lines represent group averages; shading, ±SEM. Statistics as in (C). (**F**) Left: Schematic for optogenetic measurement of laser-evoked cholinergic (ACh) release. Right: GRAB_ACh response in mPFC, averaged in 15-min bins (*n* = 10). Lines show group averages; shading, ±SEM. Statistics as in (C). n.s., not significant.

Neuromodulators play crucial roles in shaping cognition and emotion (*38*–*41*), and their dysregulation may contribute to key features of sickness behavior such as anhedonia and fatigue (*42*–*44*). We thus examined the effects of LPS on three well-known arousal-promoting neuromodulators: NE, dopamine (DA), and acetylcholine (ACh). GRAB sensor for each neuromodulator (*45*–*48*) was expressed in several brain regions, and fiber photometry recording of GRAB fluorescence was used to measure NE, DA, or ACh levels (Fig. 4, A, E, and F). We also injected a Cre-inducible AAV expressing a red-shifted channelrhodopsin (AAV5-FLEX-ChrimsonR-tdT) into the LC, ventral tegmental area (VTA), or basal forebrain (BF) of *Dbh-Cre*, *Slc6a3-Cre*, or *ChAT-Cre* mice, respectively. This allowed us to measure the release of each neuromodulator evoked by optogenetic activation of the corresponding cell type (*49*).

We first examined the effect of LPS on NE transmission. Each brief laser pulse in the LC (6 to 8 mW; 50-ms duration; applied every 60 ± 20 s) evoked a transient increase in GRAB_NE_ fluorescence (Fig. 4, B and C), and its amplitude was used to quantify evoked NE release (*49*). After LPS injection (0.4 mg/kg), evoked NE release in both the LC and medial prefrontal cortex (mPFC) were markedly diminished compared to saline control; this effect started at ~15 min postinjection and persisted throughout the 4-hour recording session [Fig. 4, C and D; repeated measures ANOVA: *F*_(19,429)_ = 2.83, *P* = 7.1 × 10^−05^; mPFC: *F*_(19,226)_ = 3.71, *P* = 9.9 × 10^−07^]. Similar effects were observed with LPS (0.1 mg/kg) or Poly (I:C) injection (20 mg/kg) (fig. S4, A and B). We then measured the calcium activity of LC neurons using jGCaMP8s. Optogenetically evoked calcium responses were also strongly reduced following LPS injection [fig. S4C; repeated measures ANOVA: *F*_(19,312)_ = 1.87, *P* = 0.02], suggesting that the reduction in evoked NE release is at least partly due to reduced excitability of LC neurons.

Next, we measured DA transmission in *Slc6a3-Cre* mice with AAV5-FLEX-ChrimsonR-tdT injected into the VTA and AAV9-hSyn-DA2h into the mPFC. In contrast to NE release, evoked DA release showed no significant change after LPS injection [Fig. 4E; repeated measures ANOVA: *F*_(1,114)_ = 2.8, *P* = 0.15]. We also examined evoked ACh release in *ChAT-Cre* mice injected with AAV5-hsyn-ACh3.0 into the mPFC and found no significant change following LPS injection [Fig. 4F; repeated measures ANOVA; *F*_(1,346)_ = 0.55, *P* = 0.46]. Together, among the three wake-promoting neuromodulators tested, evoked NE transmission was selectively suppressed by LPS injection.

### NST and PB sickness neurons regulate NE transmission

**Fig. 5.**
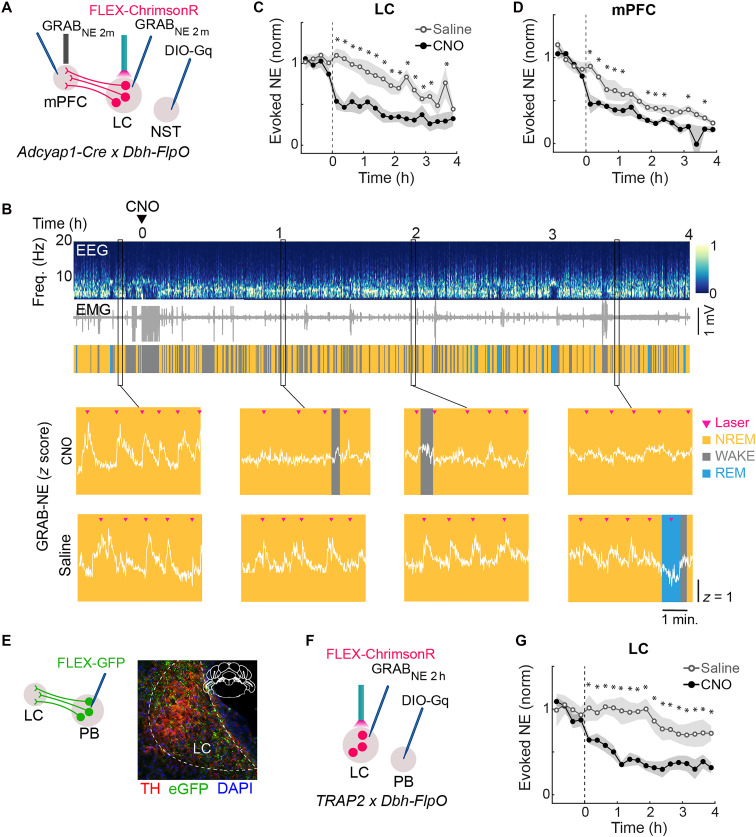
NST and PB sickness neurons regulate NE transmission. (**A**) Schematic of protocol to measure laser-evoked neurotransmitter release in LC and mPFC during chemogenetic activation of NST*^Adcyap^* neurons. (**B**) Top: Example session showing EEG, EMG, and color-coded brain states during CNO injection. Bottom: Zoomed LC GRAB_NE_ *z*-scored fluorescence traces overlaid on brain states for selected epochs before and after CNO (top) or saline (bottom) injection. Pink arrowheads indicate laser pulses to evoke release in LC. (**C**) Laser-evoked LC-NE responses during chemogenetic activation of NST*^Adcyap^* neurons, averaged in 15-min bins. Horizontal axis indicates time from CNO (black) or saline (gray) injection. Lines represent average across individual animals (*n* = 9); shading represents ±SEM. Asterisks indicate *P* values from Tukey’s post hoc test (*P < 0.05). (**D**) The same as (C) but for mPFC LC-NE evoked responses (*n* = 6). (**E**) Axonal tracing from PB^LPS-TRAP^ neurons to LC. Left: AAV8-pCAG-FLEX-eGFP was injected into the PB of TRAP2 mice. Bottom: Example fluorescence images showing LC-NE neurons (TH, red) overlapping with eGFP-labeled axons from PB (green). (**F**) Schematic for measuring laser-evoked NE release in LC during chemogenetic activation of PB^LPS-TRAP^ neurons. (**G**) Laser-evoked LC-NE responses during chemogenetic activation of PB^LPS-TRAP^ neurons, averaged in 15-min bins. Horizontal axis indicates time from CNO (black) or saline (gray) injection. Lines represent average across individual animals (*n* = 6); shading, ±SEM. Statistics as in (C).

Because activation of NST*^Adcyap1^* neurons was sufficient to recapitulate the LPS-induced increase in NREM sleep, we next asked whether activating these neurons changes NE release. *Adcyap1-Cre* mice were crossed with *Dbh-Flpo* mice to allow for simultaneous chemogenetic activation of NST*^Adcyap1^* neurons and optogenetic stimulation of LC-NE neurons. We injected Cre-inducible AAV expressing excitatory DREADD [AAV8-hM3D(G_q_)-mCherry] into the NST, Flpo-inducible AAV expressing ChrimsonR (AAV8-Ef1a-fDIO-ChrimsonR-tdT) into the LC, and AAV9-hSyn-NE2m into both the LC and mPFC of these mice (Fig. 5A). Chemogenetic activation of NST*^Adcyap1^* neurons with CNO caused a marked reduction of laser-evoked LC-NE release in both the LC and mPFC [Fig. 5, B to D, and fig. S5A; repeated measures ANOVA; LC: *F*_(19,300)_ = 2.55, *P* = 0.0005; mPFC: *F*_(1,192)_ = 2.23, *P* = 0.003], similar to the effect of LPS injection (Fig. 4, C and D, and fig. S5C).

Chemogenetic activation of PB^LPS-TRAP^ neurons also promoted NREM sleep (Fig. 3, F and G), and anterograde tracing from these neurons revealed projections to the LC, where eGFP-labeled PB^LPS-TRAP^ axons overlapped with tyrosine hydroxylase–positive LC-NE neurons (Fig. 5E). To test the effect of PB^LPS-TRAP^ neuron activation on evoked NE release, we crossed TRAP2 mice and *Dbh-Flpo* mice and injected AAV8-hM3D(G_q_)-mCherry into the PB instead of NST (Fig. 5F). Chemogenetic activation of PB^LPS-TRAP^ neurons markedly reduced evoked LC-NE release [Fig. 5G and fig. S5B, repeated measures ANOVA; *F*_(19,195)_ = 2.97, *P* = 7.2 × 10^−05^], similar to the effect of NST*^Adcyap1^* neuron activation. In control mice not expressing hM3D(G_q_) in either NST or PB, CNO had no significant effect on NE transmission [fig. S5, D to F; *F*_(1,95)_ = 0.59, *P* = 0.48]. Thus, activation of either NST or PB sickness neurons reduced evoked LC-NE release.

## DISCUSSION

In this study, we examined the neural pathway originating from the NST that promotes sleep during sickness. Using activity-dependent genetic labeling, we tagged LPS-activated NST neurons and showed that their activation drives an increase in NREM sleep, similar to the effect of LPS injection (Figs. 1 and 2 and fig. S1). These NST neurons project strongly to the PB, where sickness-activated neurons also promote NREM sleep (Fig. 3). Using GRAB sensors to monitor several wake-promoting neuromodulators (NE, DA, and ACh), we showed that NE release evoked by LC stimulation was selectively suppressed by LPS injection (Fig. 4) and by activating NST or PB sickness neurons (Fig. 5).

The NST serves as the primary hub for visceral sensory information entering the brain, integrating and relaying essential signals to regulate autonomic function and maintain homeostasis. Previous studies have shown that LPS-induced sleep increase is significantly diminished by vagotomy or NST inactivation (*23*–*27*), demonstrating the necessity of this pathway in sickness-induced sleep. Expanding on these findings, we showed that direct activation of NST sickness neurons is sufficient to promote NREM sleep (Figs. 1 and 2).

Although NST sickness neurons project to multiple brain regions (fig. S3), optogenetic activation of their axon terminals showed that only the NST → PB projection strongly promotes NREM sleep (Fig. 3). Like the NST, the PB is a key node in the central autonomic network, regulating a range of behaviors, including feeding, respiration, and thermoregulation (*50*). Previous studies have demonstrated a prominent role of the medial PB in promoting wakefulness (*51*–*53*). In our study, however, the NST sickness neurons project primarily to the lateral PB (Fig. 3A). In future studies, it would be interesting to determine the molecular identity of PB^LPS-TRAP^ neurons and their interactions with other nodes of the central autonomic network to promote sleep (*54*). In addition to the NST and PB, LPS injection activates many other brain areas involved in various aspects of sickness behavior (*22*, *24*); whether these areas contribute to sleep regulation remains to be investigated. Furthermore, while our focus was on circuits that promote sleep, sickness is accompanied by multiple behavioral changes, including loss of motivation, reduced feeding, and autonomic alterations. It will be interesting to determine how the circuits mediating these changes interact with the sleep-promoting pathway we have characterized.

Using genetically encoded biosensors to monitor NE, DA, and ACh levels, we found that peripheral immune activation selectively affects NE transmission. Evoked NE release was markedly reduced following either LPS injection or activation of NST and PB sickness neurons (Figs. 4 and 5), suggesting that these circuits promote NREM sleep in part through diminished NE transmission. In the experiment measuring evoked NE release (Fig. 4, A to D, and Fig. 5), each laser pulse caused a small but detectable increase in wakefulness and decrease in EEG delta power; LPS injection or chemogenetic activation of NST^Adcyap1^ neurons reduced not only evoked NE release but also the laser-induced increase in wakefulness and decrease in delta power (fig. S6, A to D). Notably, unlike evoked NE release, the overall NE level was elevated by LPS injection or activation of NST sickness neurons (fig. S5, A to C). This is consistent with previous studies based on microdialysis (*55*–*58*), and it may be part of a negative feedback mechanism that helps to dampen inflammation (*59*–*62*). The increase in overall NE levels is likely to contribute to sickness-induced suppression of REM sleep (*19*, *63*–*65*), although the increase in NREM sleep seems to contradict the wake-promoting effect of LC-NE activity (*66*, *67*). On the other hand, accumulating evidence suggests that while transient NE increases promote arousal, sustained NE elevation may enhance NREM sleep (*64*, *68*–*70*). An increase in overall NE levels induced by the NE reuptake blocker desipramine also led to a decrease in evoked NE release (fig. S6, E to I), likely due to presynaptic α2 adrenergic receptors expressed on LC-NE axon terminals (*71*–*73*). This suggests that these two opposing changes in LC-NE activity are closely linked. A recent study showed that sickness-induced pathogen-avoidance behavior in *Caenorhabditis elegans* depends on suppression of tyramine/octopamine signaling, which is often considered the invertebrate counterpart of vertebrate NE signaling (*74*).

We showed that both LPS and Poly(I:C) injections promote NREM sleep (fig. S1) and reduce evoked NE transmission (Fig. 4, A to D, and fig. S4, A and B). These manipulations, which mimic bacterial and viral infections, may promote sleep through shared mechanisms, for example, via proinflammatory cytokines such as tumor necrosis factor and interleukin-1β. These cytokines have been shown to activate subsets of NST neurons (*28*) and promote NREM sleep by activating sleep-promoting neurons and/or inhibiting wake-promoting neurons (*10*, *75*). In addition to the neural pathway via the NST, cytokines injected into the brain can also promote sleep by directly inhibiting wake-promoting serotonergic neurons in the dorsal raphe (*10*, *76*–*78*).

In summary, we have identified an NST➔PB pathway that mediates sickness-induced NREM sleep, in part by modulating LC-NE transmission. Besides peripheral immune activation, LC-NE signaling is also regulated by microglia, the brain’s resident immune cells (*79*). As a potent wake-promoting neuromodulator regulated by both neuronal and nonneuronal mechanisms, the NE system is well positioned to link the body’s need for recovery from sickness to the homeostatic regulation of sleep drive.

## MATERIALS AND METHODS

### Animals

All procedures were performed in accordance with the protocol approved by the Animal Care and Use Committee at the University of California, Berkeley (AUP-2016-06-8860-3). Adult (6 to 12 weeks old) male and female mice were used for all experiments. Mice were kept on a 12:12 light:dark cycle (lights on at 07:00 a.m. and off at 07:00 p.m.) with free access to food and water. The animals were housed in temperature- and humidity-controlled environment (20° to 25.6°C; 40 to 70% humidity). After virus injections and surgical implantation of EEG/EMG electrodes and optical fibers, the mice were individually housed to prevent damage to the implant before experiments. The experiments were conducted at least 2 weeks after surgery. Mice used here include as follows: TRAP2: Jackson strain # 030323; *Adcyap1*-Cre: Jackson strain # 030155; Dbh-Cre: B6.FVB(Cg)-Tg(Dbh-Cre)KH212Gsat/Mmucd, MMRRC: 036778-UCD; Dbh-Flpo: Jackson strain # 033952; *Slc6a3*-Cre: Jackson strain # 006660; CHAT-Cre: Jackson strain # 06410; Ai14: Jackson strain #007914; and EGFP-L10a: Jackson strain #024750.

### LPS administration

LPS from *Escherichia coli* (O111:B4, Sigma-Aldrich: L2630) was reconstituted in saline (1 mg ml^−1^) and frozen into single-use aliquots. Further dilutions in saline to the experimental dose were prepared before each experiment. All doses were delivered by intraperitoneal injection during the light phase [Zeitgeber time (ZT) 2 to 7]. For most experiments, LPS was administered only once to avoid desensitization with repeated exposure. For G_i_ inhibition experiments using TRAP2 mice (Fig. 1, I to K), the number of LPS exposures was controlled between G_i_ and mCherry by injecting both groups of animals with LPS and tamoxifen for TRAP induction and once again with LPS for experiments.

### Poly(I:C) administration

Poly(I:C) (Fisher Scientific: 42-875-0) was diluted in sterile saline to 1 mg/ml and heated at 65°C for 10 min. Stock solutions were allowed to cool before storage at −20°C until use. It was delivered by intraperitoneal injection during the light phase (ZT 2 to 6).

### Desipramine administration

Desipramine (Fisher Scientific: 30-675-0) was diluted in sterile saline to 1 mg/ml and stored at −20°C until use. It was delivered by intraperitoneal injection during the light phase (ZT 2 to 6).

### TRAP induction

The 4-OHT was prepared based on K. Deisseroth’s laboratory protocol. For LPS-TRAP, 4-OHT (2 mg/ml in saline with 2% Tween-80, 20 mg/kg) and LPS (0.04, 0.4, 1 mg/kg) were coinjected intraperitoneally during the light phase (ZT 2 to 7). Mice were given at least 7 days to allow for expression before beginning experiments. To measure the overlap between LPS-TRAP and LPS-Fos, mice were euthanized and perfused 1 to 2 hours after LPS injections.

### Surgical procedures

Adult mice (6 to 12 weeks old; male and female) were anesthetized with isoflurane (3% induction, 1.5% maintenance) and placed on a stereotaxic frame. Buprenorphine [0.1 mg/kg, subcutaneously (sc)] and meloxicam (10 mg/kg, sc) were injected before surgery. Lidocaine (0.5%, 0.1 ml, sc) was injected near the target incision site. Body temperature was stably maintained throughout the procedure using a heating pad. After asepsis, the skin was incised to expose the skull and overlying connective tissue was removed.

For EEG and EMG recordings, a reference screw was inserted into the skull on top of the left cerebellum. EEG recordings were made from two screws on top of the left and right cortex at −3.5 [anteroposterior (AP)] ± 3.5 mm [mediolateral (ML)]. Two EMG electrodes were inserted into the neck musculature. Insulated leads from the EEG and EMG electrodes were soldered to a pin header, which was secured to the skull using dental cement.

Virus injections were performed as above, but a craniotomy was made on top of target regions (see below for coordinates) and 50 to 200 nl of the virus was injected using a Nanoject II (Drummond) and a glass micropipette. For optogenetic and fiber photometry experiments, optic fibers (1.25-mm ferrule, 200 μm Core, 0.39 numerical aperture) were stereotactically inserted and secured to the skull using dental cement. All experiments were performed at least 2 weeks after surgery to allow for virus expression and animal recovery.

The following stereotaxic coordinates were used for virus injections and optogenetic cannula placement. Unless otherwise noted, coordinates are listed relative to bregma.

NST: −7.3 AP, 0.25 ML, −5.0 dorsoventral (DV)

PVT: −0.4 AP, 0 ML, −3.6 DV

vlPAG: −4.9 AP, 0.6 ML, −2.7 DV

VTA: −3.2, 0.5, 4.1 from the dura

mPFC: +2.1 AP, 0.3, −1.6 DV

BF: +0.1 AP, 1.5 ML, 5.3 DV

LC: −5.5 AP, 0.9 ML, −3.8 to −3.2 DV. For LC, 50 nl was injected every 0.2 mm at multiple depths. Fibers were placed at −3.65 DV.

PB: −4.9 AP, 1.5 ML, −3.7 DV

### Viruses

AAV5-hSyn-DIO-hM3D(G_q_)-mCherry (#44361), AAV5-hSyn-DIO-hM4D(G_i_)-mCherry (# 44362), AAV8-pCAG-FLEX-eGFP-WPRE (# 51502), AAV9-syn-FLEX-jGCaMP8s-WPRE (#162377), and AAV5-hSyn-FLEX-ChrimsonR-tdT (# 62723) were obtained from Addgene. AAV5-EF1a-DIO-SSFO-mCherry was obtained from the University of North Carolina (UNC) vector core. GRAB sensors AAV9-hSyn-NE2m, AAV5-hsyn-ACh3.0(ACh4.3), and AAV9-hSyn-DA2h(DA4.3) were obtained from WZ Biosciences.

### Polysomnographic recordings

Behavioral experiments were carried out in home cages placed in sound-attenuating boxes during the light phase (ZT2 to ZT12) at ambient room temperature (~20° to 25.6°C). EEG/EMG electrodes were connected to flexible recording cables via a mini-connector. EEG/EMG signals were acquired using a TDT PZ5 amplifier and Synapse software, with a band-pass filter of 0.3 to 500 Hz and sampling rate at 1017 Hz. Spectral analysis was carried out using fast Fourier transform, and brain states were classified as described previously [wake: desynchronized EEG and high EMG activity; NREM: synchronized EEG with high-amplitude, low-frequency (1 to 4 Hz) activity and low EMG activity; REM: high EEG power at theta frequencies (6 to 9 Hz) and low EMG activity]. The classification was determined using 5-s bins and with a custom-written graphical user interface (programmed in MATLAB, MathWorks).

### Fiber photometry recording

Fiber photometry recording was performed using TDT RZ10x real-time processor. Fluorescence elicited by 405- and 465-nm light-emitting diodes were filtered through the dichroic mini cube (Doric lenses) and collected with an integrated photosensor on the RZ10x. Signals were demodulated and preprocessed using the TDT Synapse software collected at a sampling frequency of 1017 Hz.

### Optogenetics

For NST terminal stimulation experiments (Fig. 3), we applied 5 s of constant blue light (6 mW) from a laser diode (473 nm, RWD Life Science) every 20 min (±5 min of random jitter) for the duration of the recording session. Laser power was measured from the fiber tip before the start of each experiment to ensure consistency between experimental conditions. Each experimental session lasted for 4 hours, and each animal was tested for four to eight sessions.

To measure evoked NE release, a patch cable from the 635 red laser diode (RWD Life Science) was connected to the dichroic mini-cube (Doric) to enable simultaneous optogenetic laser stimulation and fiber photometry from the same fiber tip. We applied 50 ms of red light (6 to 8 mW) every 60 s (±20 s of random jitter) for the duration of each 5-hour recording session. Laser power was measured from the fiber tip before the start of each experiment to ensure consistency between experimental conditions.

### Immunohistochemistry and FISH

Mice were deeply anesthetized and transcardially perfused with 0.1 M phosphate-buffered saline (PBS) followed by 4% paraformaldehyde in PBS. For fixation, samples were kept overnight in 4% paraformaldehyde. Samples were then placed in a 30% sucrose solution for 36 to 48 hours. After embedding and freezing, the brains were sectioned into 30-μm (FISH samples) or 50-μm (for other immunohistochemistry) coronal slices. For immunohistochemistry, the brain slices were washed using PBS three times, permeabilized using PBST (0.3% Triton X-100 in PBS) for 30 min, and then incubated with blocking solution (5% normal goat serum or normal donkey serum in PBST) and primary antibody solution (1:500 in PBST) overnight at 4°C. Antigen retrieval pretreatment was performed for c-Fos staining (Synaptic Systems, 226 008). The next day, the slices were washed with PBS and incubated with appropriate secondary antibodies for 2 hours at room temperature or overnight at 4°C. FISH was performed using RNAscope Multiplex Fluorescent Assays V2 according to the manufacturer’s instructions (Advanced Cell Diagnostics). Fluorescence images were taken using a fluorescence microscope (Keyence BZ-X710) or a high-throughput slide scanner (Nanozoomer-2.0RS, Hamamatsu).

### Quantification and statistical analysis

For fiber photometry experiments, the 405-nm channel was used to correct nonspecific, calcium-independent changes in fluorescence, e.g., movement artifacts. Each channel (465 and 405 nm) was first fit with a single exponential to remove the baseline change due to bleaching. The 405-nm signal was then fit to the 465-nm signal using a least-squares linear fit method (*80*) and then subtracted from the 465-nm signal. The resulting signals were then converted to *z* scores based on the mean and SD of the entire imaging session.

For changes in overall level, corrected photometry signals for each session type (saline, CNO) were averaged into 15-min bins and subtracted for each mouse (CNO - saline). This difference was then averaged across mice to assess changes in overall level (figs. S5 and S6). For laser-evoked release, 465-channel signals were *z* scored and normalized to the mean of the first hour of the 5-hour session. Evoked response for each pulse was calculated as the difference between the time period 1 s after laser onset and the 1 s before laser onset. Evoked responses were normalized to the first hour of the recording session and binned every 15 min. To control for differences in evoked release across brain states, we restricted all analyses to periods of NREM sleep.

Statistical analyses were conducted using MATLAB and R. Most analyses used two-way repeated measures mixed ANOVAs implemented in R. We specified random slopes and intercepts models and included mouse/subject as a random covariate using the lme4 package. Treatment (CNO, LPS, poly(I:C) or saline) and time (binned by hours or minutes of the recording session) were included as fixed effects. Following a significant main effect or interaction, we conducted post hoc analyses between groups across time. Reported post hoc analyses are Tukey’s honest significant difference test and were conducted using the lsmeans or emmeans package in R. All statistical comparisons are conducted on animal averages [i.e., each animal has one observation per level(s) of the independent variable].
