# Supplementary material for: Brainstem circuit for sickness-induced sleep
Source: Sci Adv. 2025 Dec 10;11(50):eady0245. doi: 10.1126/sciadv.ady0245 (PMC12693964; doi:10.1126/sciadv.ady0245)
Supplement: Supplementary file 1 — Figs. S1 to S6 [file sciadv.ady0245_sm.pdf]

Science Advances
AAAS

Supplementary Materials for

Brainstem circuit for sickness-induced sleep

Dana Darmohray *et al.*

Corresponding author: Yuanyuan Yao, yyyao@smart.org.cn; Yang Dan, danyang@smart.org.cn

Sci. Adv. **11**, eady0245 (2025)
DOI: 10.1126/sciadv.ady0245

This PDF file includes:

Figs. S1 to S6

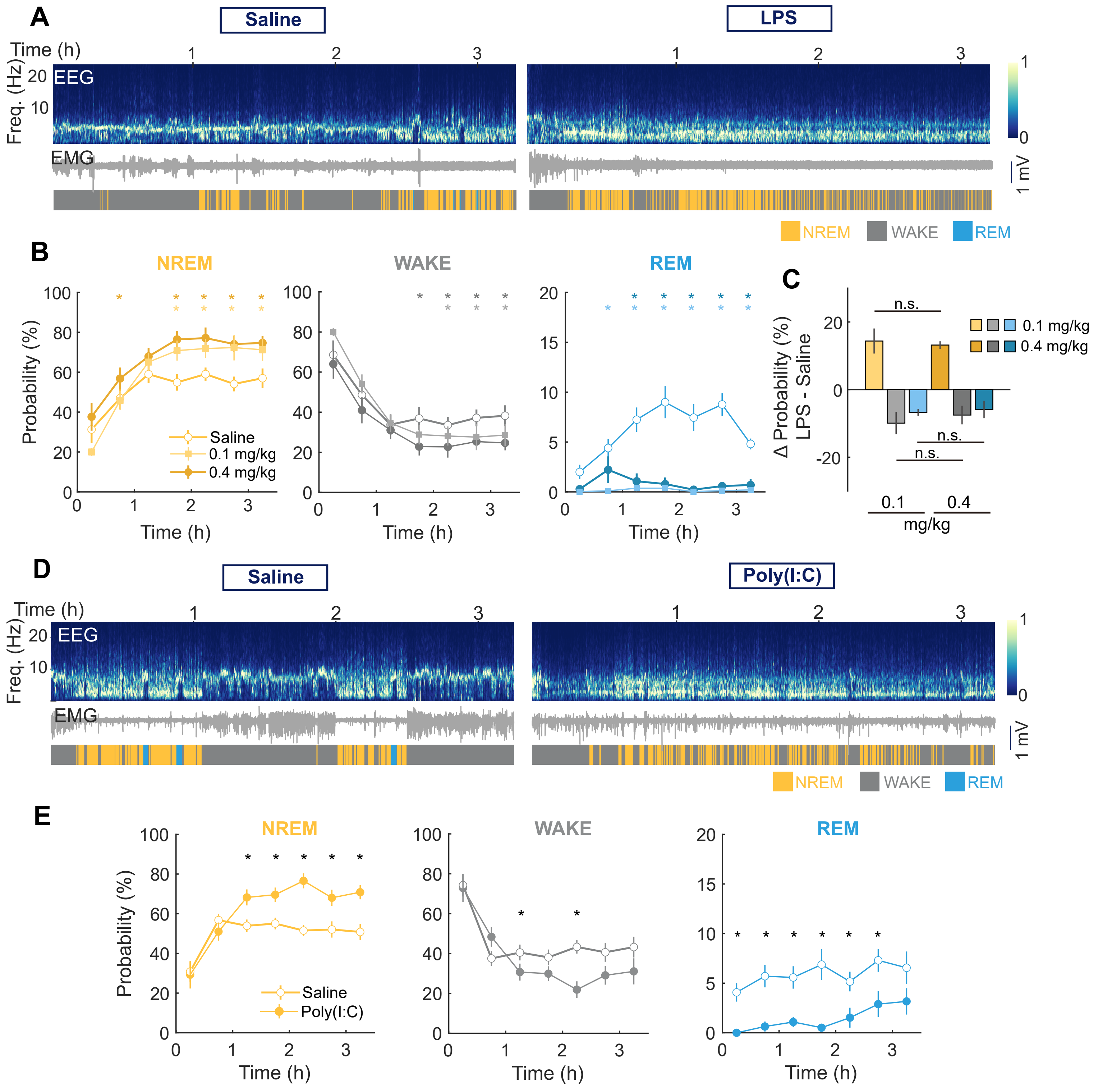
A
Saline
LPS
Time (h)
Freq. (Hz)
EEG
EMG
1 mV
NREM
WAKE
REM
B
NREM
WAKE
REM
Probability (%)
Time (h)
Saline
0.1 mg/kg
0.4 mg/kg
C
n.s.
Δ Probability (%)
LPS - Saline
0.1 mg/kg
0.4 mg/kg
0.1
0.4
mg/kg
D
Saline
Poly(I:C)
E
Saline
Poly(I:C)

Fig. S1. LPS and poly (I:C) injection promote NREM sleep and suppress REM sleep
(A) Example showing effects of LPS (0.4 mg/kg) injection on brain states. EEG, EMG, and color-coded brain states are shown for saline (*left*) and LPS (*right*) injection across an over 3-h recording.

(B) Average changes in NREM, wake and REM following saline (open circles) or LPS injection (0.1 or 0.4 mg/kg, solid circles). Asterisks indicate p values from Tukey's HSD test ($^*p < 0.05$). Error bars represent ±SEM.

(C) Differences in brain states between saline and LPS (0.1 mg/kg, lighter colors, n = 9; 0.4 mg/kg, darker colors, n = 10) injections. No significant difference was observed between two doses (independent samples *t*-test; WAKE: $t_{(16)} = 0.80$, $p = 0.43$; NREM: $t_{(16)} = -0.84$, $p = 0.41$, REM: $t_{(16)} = -0.47$, $p = 0.64$).

(D) Example showing effects of Poly (I:C) (20 mg/kg) injection on brain states. EEG, EMG, and color-coded brain states for saline (*left*) and poly(I:C) (*right*) are shown across an over 3-h recording.

(E) Average changes in NREM, wake and REM following saline (open circles) or Poly (I:C) injection (solid circles, n = 14). Horizontal axis represents time after injection. Statistics as in **(B)**.

A

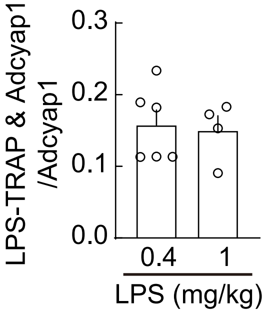

Fig. S2. Ratio of NST Adycyap1 neurons co-labeled by LPS-TRAP

(A) Percentage of NST Adycyap1 neurons co-labeled by LPS-TRAP. Open circles represent individual samples.

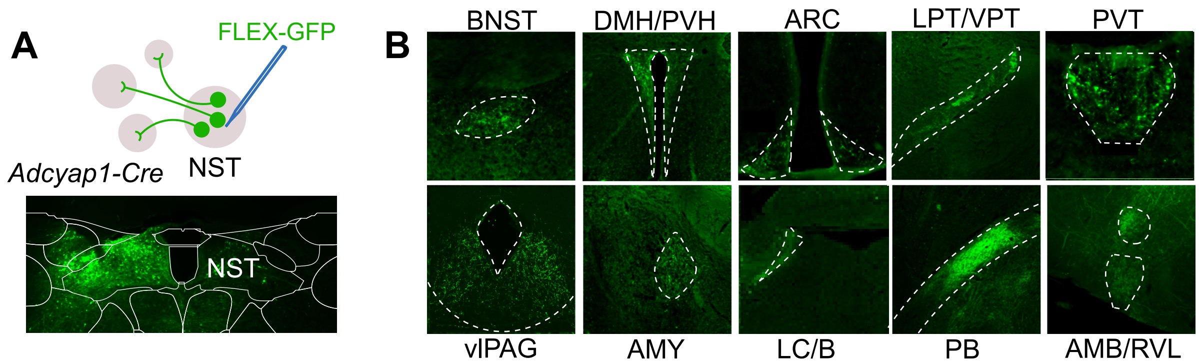
A
FLEX-GFP
Adcyap1-Cre
NST
NST
B
BNST
DMH/PVH
ARC
LPT/VPT
PVT
vlPAG
AMY
LC/B
PB
AMB/RVL

Fig. S3. Projections of NST^{Adcyap} neurons

(A) *Top:* Schematic of anterograde tracing from NST^{Adcyap} neurons. AAV8-pCAG-FLEX-eGFP was injected into the NST of *Adcyap1*-Cre mice to label axonal terminals. *Bottom:* Example of eGFP-labeled NST^{Adcyap} neurons.

(B) Projection targets of NST^{Adcyap} neurons. eGFP-labeled axon terminals were observed in 14 brain regions, including the bed nucleus of the stria terminalis (BNST), the dorsomedial and paraventricular hypothalamus (DMH/PVH), the arcuate nucleus of the hypothalamus (ARC), the lateral posterior and ventroposterior thalamus (LPT/VPT), the periventricular thalamus (PVT), the periaqueductal gray (PAG), the amygdala (AMY), the locus coeruleus and the Barrington's nucleus (LC/B), the parabrachial nucleus (PB), the nucleus ambiguous and the rostral ventrolateral medulla (AMB/RVLM).

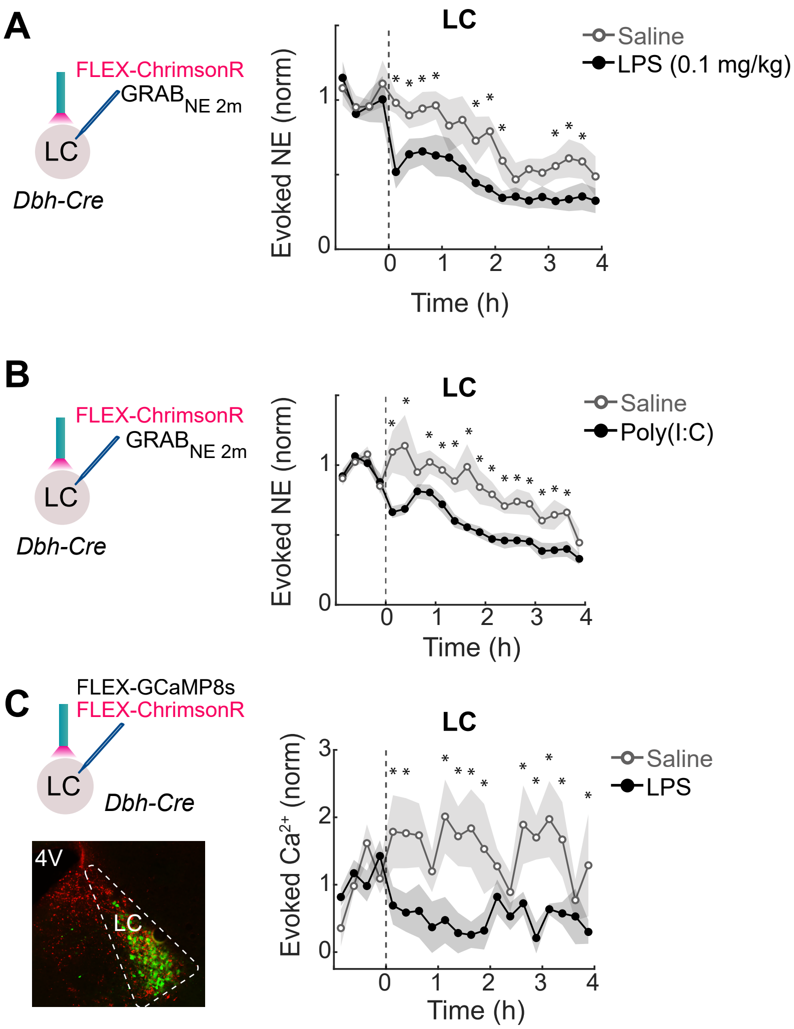

A
FLEX-ChrimsonR
GRAB NE 2m
LC
Dbh-Cre
LC
Saline
LPS (0.1 mg/kg)
Evoked NE (norm)
Time (h)
B
FLEX-ChrimsonR
GRAB NE 2m
LC
Dbh-Cre
LC
Saline
Poly(I:C)
Evoked NE (norm)
Time (h)
C
FLEX-GCaMP8s
FLEX-ChrimsonR
LC
Dbh-Cre
4V
LC
LC
Saline
LPS
Evoked Ca2+ (norm)
Time (h)

Fig. S4. Low-dose LPS or Poly (I:C) injection reduces LC-NE evoked responses. LPS injection reduces laser- evoked calcium responses of LC.

(A) *Left:* Schematic of protocol to measure laser-evoked release in NE neurons**.** *Right:* Laser-evoked $GRAB_{NE}$ responses in LC (n = 5), averaged in 15-min bins following LPS (black, 0.1 mg/kg) or saline (gray) injection. LPS significantly reduced evoked NE release (repeated measures ANOVA: $F_{(1,195)} = 73.63$, $p = 2.9e\text{-}15$). Lines represent mean responses across mice; shading, ±SEM. Asterisks indicate significance by Tukey's HSD test ($^{*}p < 0.05$).

(B) *Left:* Schematic as in **(A).** *Right:* Laser-evoked $GRAB_{NE}$ responses in the LC following Poly (I:C) (black, 20 mg/kg) or saline (gray) injection (n = 8). Poly(I:C) significantly reduced evoked NE release (repeated measures ANOVA: $F_{(19,390)} = 1.71$, $p = 0.03$). Lines and shading as in **(A)**.

(C) *Top left:* Schematic for measuring laser-evoked calcium responses in LC neurons**.** *Bottom left***:** Example showing LC-NE neurons co-expressing ChrimsonR (tdTomato) and jGCaMP8s. *Right:* Laser-evoked calcium responses in the LC (n = 9) following LPS (black, 0.4 mg/kg) or saline (gray) injection, averaged in 15-min bins. Data are shown as in **(A)**.

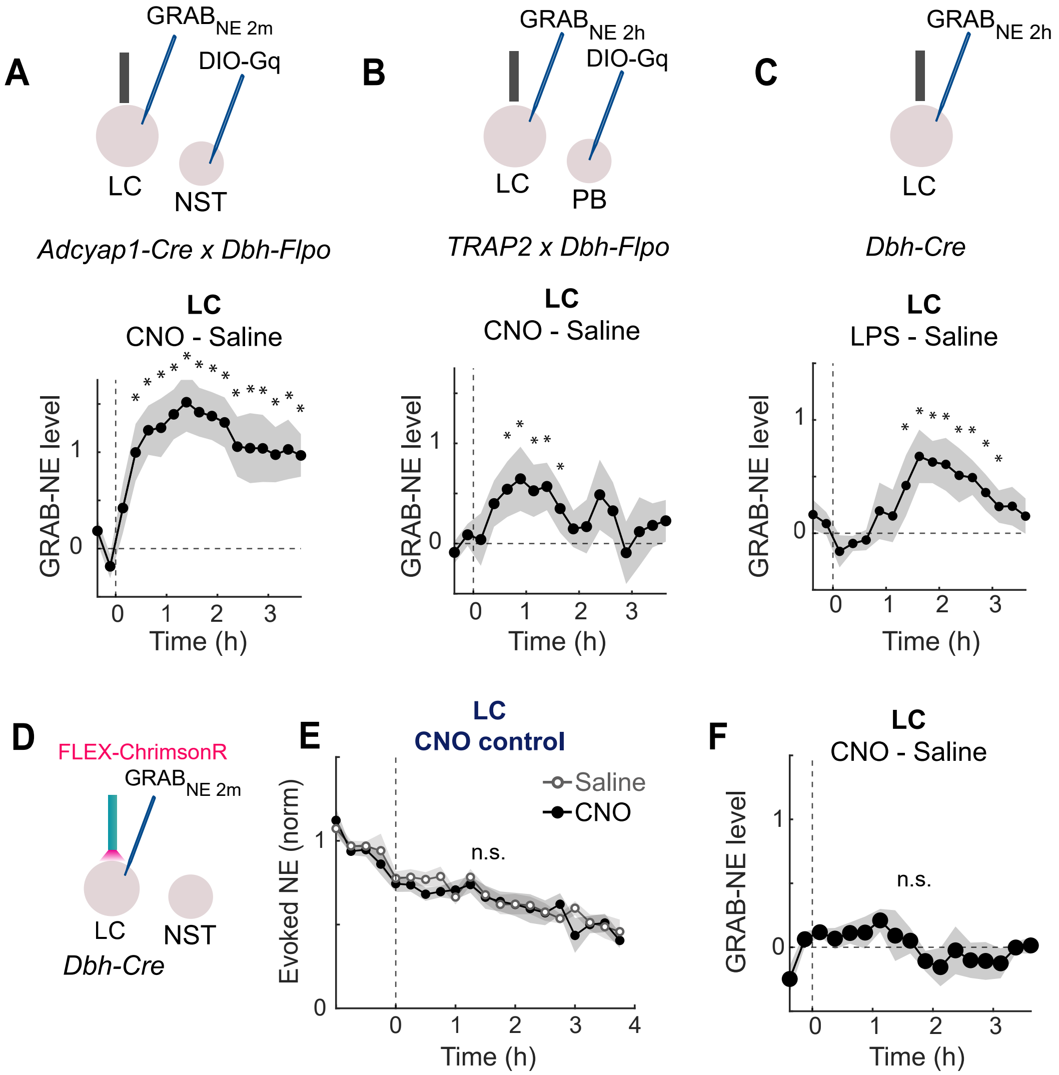

A
GRAB NE 2m
DIO-Gq
LC
NST
Adcyap1-Cre x Dbh-Flpo
LC
CNO - Saline
GRAB-NE level
Time (h)
B
GRAB NE 2h
DIO-Gq
LC
PB
TRAP2 x Dbh-Flpo
LC
CNO - Saline
GRAB-NE level
Time (h)
C
GRAB NE 2h
LC
Dbh-Cre
LC
LPS - Saline
GRAB-NE level
Time (h)
D
FLEX-ChrimsonR
GRAB NE 2m
LC
NST
Dbh-Cre
E
LC
CNO control
Saline
CNO
n.s.
Evoked NE (norm)
Time (h)
F
LC
CNO - Saline
n.s.
GRAB-NE level
Time (h)

Fig. S5. Changes in overall NE level induced by LPS or chemogenetic activation of sickness neurons.
(A) *Top***:** Schematic of recording LC NE levels during chemogenetic activation of NST^{Adcyap} neurons. *Bottom***:** Average difference (CNO - Saline; n = 9) of LC $GRAB_{NE}$ z-scored fluorescence traces, binned in 15-min intervals. CNO significantly increased overall NE level (repeated measures ANOVA: $F_{(16,297)} = 1.94$, $p = 0.02$). Horizontal axis indicates time from CNO injection. Shading, ±SEM. Asterisks indicate significance by Tukey's HSD test ($^{*}p < 0.05$).

(B) *Top***:** Schematic of recording LC NE levels during chemogenetic activation of $PB^{LPS\text{-}TRAP}$ neurons. *Bottom***:** Average difference (CNO - Saline; n = 6) of LC $GRAB_{NE}$ traces binned in 15min intervals. CNO significantly increased NE overall level (repeated measures ANOVA: $F_{(1,165)} = 41.59$, $p = 1.2e\text{-}09$). Data are shown as in **(A)**.

(C) *Top***:** Schematic of recording LC NE levels following LPS injection (0.4 mg/kg). *Bottom*: Average difference (LPS - Saline; n = 12) of LC $GRAB_{NE}$ traces binned in 15-min intervals. LPS significantly increased NE overall level (repeated measures ANOVA: $F_{(16,363)} = 2.50$, $p = 0.001$). Data are shown as in **(A)**.

(D) Schematic of measuring laser-evoked NE release in control mice lacking hM3D (Gq) expression in the NST.

(E) Laser-evoked $GRAB_{NE}$ responses in the LC of control mice (n = 6), binned in 15-min intervals following CNO (black) or saline (gray) injection. Lines represent average across mice; shading represent ±SEM. Data are analyzed as in **(A)**.

(F) Average difference (CNO - Saline; n = 6) of LC $GRAB_{NE}$ traces binned in 15-min intervals. CNO alone (0.3 mg/kg) did not change overall NE level (repeated measures ANOVA: $F_{(1,80)} = 0.0009$, $p = 0.97$). Shading represents ±SEM. Asterisks indicate significance from paired samples *t*-tests($^{*}p < 0.05$, $^{**}p < 0.01$, $^{***}p < 0.001$).

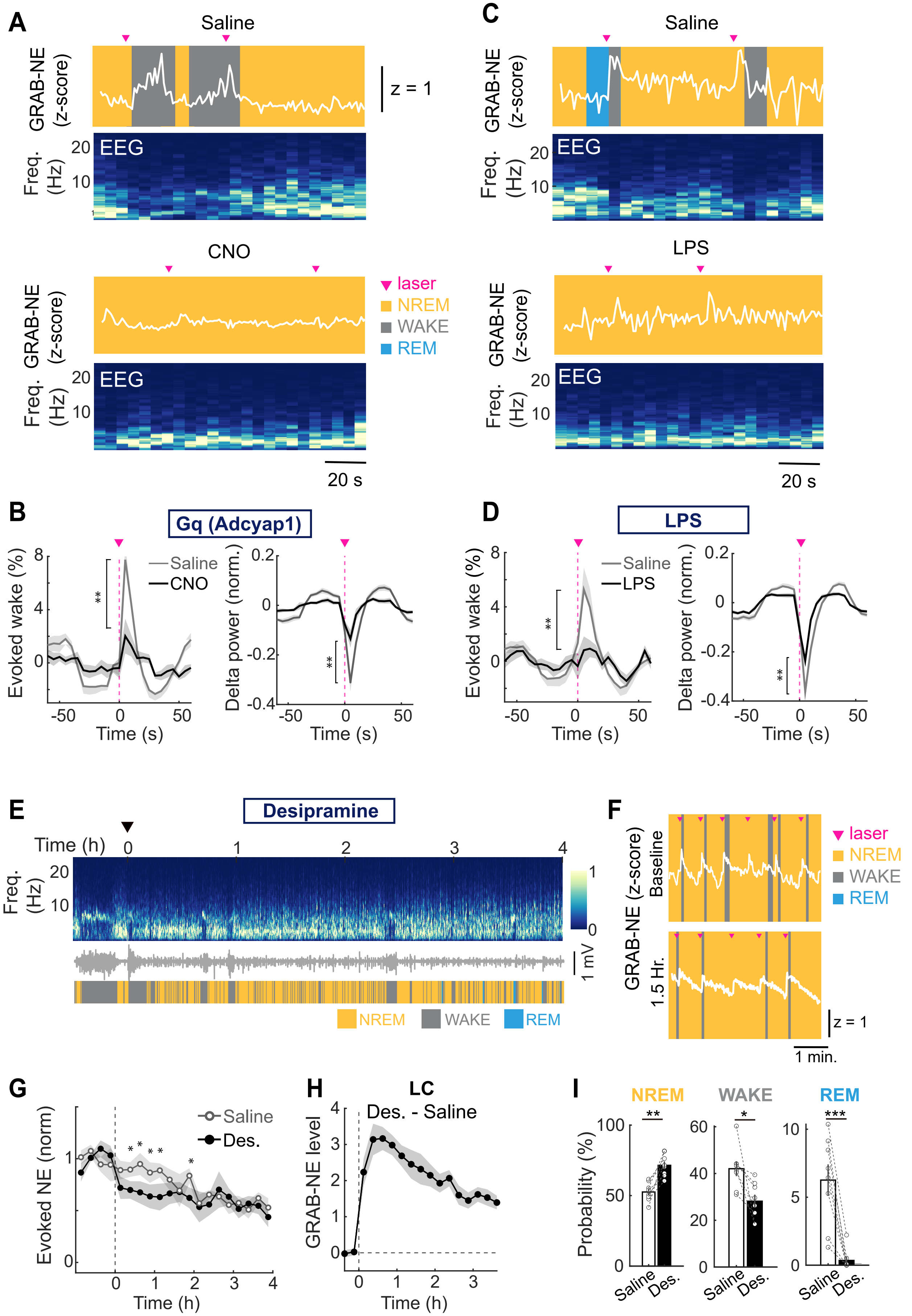

A
Saline
GRAB-NE (z-score)
z = 1
Freq. (Hz)
EEG
CNO
laser
NREM
WAKE
REM
20 s
C
LPS
B
Gq (Adcyap1)
Evoked wake (%)
Saline
CNO
Delta power (norm.)
Time (s)
D
E
Desipramine
Time (h)
1 mV
F
Baseline
1.5 Hr.
1 min.
G
Evoked NE (norm)
Des.
H
LC
Des. - Saline
GRAB-NE level
I
Probability (%)

Fig. S6. LPS or activation of NST sickness neurons reduces LC-NE evoked responses and evoked wakefulness, while NE-reuptake inhibitor reduces LC-NE evoked responses and enhances NREM sleep.

(A) *Top:* Laser-evoked NE release and brain state changes following optogenetic stimulation (50 ms) of LC *Dbh*+ neurons (pink arrows). Examples show saline (top) or CNO (bottom) injection in *Adcyap1-Cre x Dbh-FlpO* mice expressing hM3D(Gq) in NST^{Adcyap} neurons.

(B) Average (±SEM) laser-evoked changes in wakefulness during chemogenetic activation of NST^{Adcyap} neurons (paired *t*-test: $t_{(8)} = 2.88, p = 0.05$). *Right:* Same as B but for normalized delta power (paired *t*-test: $t_{(8)} = -3.02, p = 0.02$). Pink arrow, laser onset.

(C) Similar to (A), but following saline or LPS (0.4 mg/kg) injection.

(D) Similar to (B), but for saline or LPS injection (n = 12; paired *t*-test: $t_{(11)} = 3.21, p = 0.01$). *Right:* Corresponding changes in laser-evoked delta power (paired *t*-test: $t_{(11)} = -7.43, p = 0.00001$).

(E) Example of NE-reuptake inhibitor, desipramine (Des., 20 mg/kg). EEG, EMG, and color-coded brain states across a 4-h recording.

(F) LC $GRAB_{NE}$ z-scored fluorescence traces on brain-state labels during saline or Des. sessions. Pink arrowheads, laser onset.

(G) Average (±SEM) laser-evoked $GRAB_{NE}$ responses for LC (n = 9), shown in 15-min bins following Des. or saline injection (repeated measures ANOVA: $F_{(1,312)} = 10.12, p = 0.002$). Tukey's HSD: $*p < 0.05$.

(H) LC-NE overall level differences (Des. - saline) binned in 15-min intervals (repeated measures ANOVA: $F_{(16,264)} = 15.24, p = 2.2e\text{-}16$). Data are shown as in **(G)**.

(I) Brain state probability (mean ±SEM) following saline and Des. groups. Desipramine increased NREM and reduced wake and REM (paired *t*-tests: NREM: $t_{(7)} = -5.17, p = 0.001$; WAKE: $t_{(7)} = 2.98, p = 0.02$; REM: $t_{(7)} = 5.82, p = 0.00006$). Circles, individual mice.
